# Retro-Malleolar Z-Plasty of Flexor Hallucis Longus Tendon in Post-Traumatic Checkrein Deformity: A Case Series and Literature Review

**DOI:** 10.3390/medicina58081072

**Published:** 2022-08-10

**Authors:** Chiara Polichetti, Tommaso Greco, Michele Inverso, Giulio Maccauro, Fabrizio Forconi, Carlo Perisano

**Affiliations:** 1 Department of Ageing, Neurosciences, Head-Neck and Orthopedics Sciences, Orthopedics and Trauma Surgery Unit, Fondazione Policlinico Universitario Agostino Gemelli IRCCS, 00168 Rome, Italy; 2 Orthopedics and Trauma Surgery, Università Cattolica del Sacro Cuore, 00168 Rome, Italy; 3 Casa Di Cura Villa Stuart, Via Trionfale 5952, 00135 Rome, Italy

**Keywords:** checkrein deformity, foot deformities, tibial fracture, flexor hallucis longus, claw toe deformity, Z-plasty

## Abstract

Checkrein deformity (CD) is a dynamic deformity of the hallux characterized by flexion contracture of the interphalangeal (IF) joint and extension contracture of the metatarsophalangeal (MTP) joint, worsened by ankle dorsiflexion. It is due to post-traumatic or ischemic retraction of the long hallux flexor tendon (FHL) following soft tissue trauma, leg fractures, ankle fractures and, more rarely, calcaneal or talar fractures. Diagnosis is essentially clinical, associated with imaging, to rule out unrecognized causes and evaluate fracture healing process. Few cases are reported in literature without univocal treatment. *Background and Objectives*: To analyze clinical and functional outcomes in patients with CD treated with release and retro-malleolar Z-plasty lengthening of FHL tendon. *Materials and Methods*: Patients diagnosed with CD treated with retro-malleolar (at tarsal tunnel) Z-plasty lengthening of the FHL tendon between January 2016 and August 2020 were included. Clinical and functional outcomes were collected on admission and post-surgery and analysed retrospectively. Patients with a minimum follow-up of 18 months were included. *Results*: A total of 14 patients, with mean age of 37.4 years old, with CD diagnosis were included in the study. All patients were suffering from post-traumatic CD and the mean time from trauma to onset of deformity was of 7 months (range 1–12). At a mean follow-up of 31.8 months (range 18–48) we found a significant improvement (*p* < 0.05) in terms of pain relief (VAS), function (AOFAS score) and ROM of the IP and MTP hallux joints. No recurrence, loss of strength, nerve injury or tarsal tunnel syndrome were observed. No patient required revision surgery. *Conclusions*: In this case series the retro-malleolar FHL tendon Z-plasty proved to be a suitable option for CD correction, allowing a good clinical and functional recovery.

## 1. Introduction

Checkrein deformity (CD) is a rare claw toe dynamic deformity of the hallux, due to retraction of the flexor hallucis longus (FHL) tendon [1]. Usually it occurs after tibial, leg [1,2,3,4,5,6,7,8,9] and in ankle [8,10,11,12,13] fractures but in the literature cases have also been reported following calcaneus fractures [14], talar fracture [15,16], removal of the fibula for bone graft [10,17,18], subtalar dislocation [19] and soft tissue trauma with compartment syndrome [5,20].

Considering the few cases found and analyzed in the literature, it is difficult to establish the prevalence of this deformity, partly because it often remains unrecognized, but it should always be considered in patients who have suffered such trauma.

CD etiopathogenesis is debated in the literature, where it has been suggested that the deformity is the result of entrapment of the FHL in scar tissue or newly formed bone callus at the fracture site [14], but others feel that it results from contracture of the muscles after subclinical leg compartment syndrome [5].

**Figure 1:**
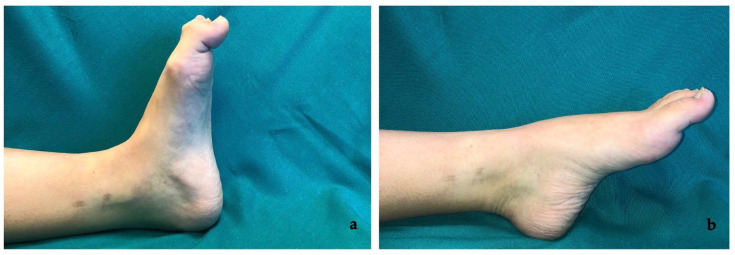
Pre-surgery clinical images of case no.6 in the series. (**a**) Checkrein Deformity (CD) at dorsiflexion and (**b**) resolution of the deformity with ankle plantar flexion.

The clinical presentation is a flexion contracture of the hallux interphalangeal (IP) joint with mild extension contracture of the metatarsophalangeal (MTP) joint, that worsens with passive dorsi-flexion of the ankle [1] (Figure 1, see Supplementary Material Video S1). Considering the interconnections between FHL and FDL tendons at the master knot of Henry, occasionally deformities of the other toes are associated [1,2,5,6,8,17,18,20].

Diagnosis is essentially clinical. Radiography can be used to investigate the fracture healing process, while Computed Tomography (CT) or Magnetic Resonance Imaging (MRI) may be helpful in cases with unknown etiology and pathogenesis to identify any unrecognized causes [8,18,21,22,23].

Available literature shows that treatment is in almost all cases surgical. Only in a single case report were physical therapy and rehabilitation used to treat CD, obtaining pain relief and improvement in the patient′s walking ability [17]. Different surgical procedures have been proposed: simple FHL tendon adherence release at fracture site [11,12], release associated to FHL tendon Z-plasty lengthening at fracture site [5], midfoot Z-plasty tendon lengthening without release [10] and retro-malleolar (at tarsal tunnel) FHL tendon Z-plasty [1,15,20]. In all these cases, surgery was followed by physical rehabilitation to recover and maintain range of motion of ankle and toe joints.

This study aims to analyze clinical and functional outcomes obtained with release and retro-malleolar (at tarsal tunnel) Z-plasty lengthening of FHL tendon in 14 patients with post-traumatic CD.

## 2. Materials and Methods

A retrospective observational study according to the STROBE guidelines [24] (Appendix A) was conducted.

From January 2016 to August 2020, all patients consecutively admitted to the Orthopedic and Trauma Surgery Unit of our Institution with clinical diagnosis of CD were considered for inclusion in the study. Inclusion criteria were patients with CD diagnosis, surgical treatment with retro-malleolar FHL tendon Z-plasty and follow-up of at least 18 months.

Demographic characteristics, clinical features and surgical data of the patients were retrospectively collected through clinical and outpatient records. Follow-up was performed through outpatient clinical evaluation at 1, 3, 6, 12, 18, 24 and 48 months after surgery. During the follow-up, primary outcomes in terms of pain relief (Visual Analogue Scale, VAS) [25], function recovery (American Orthopedic Foot and Ankle Society Score, AOFAS) [26], hallux IP and MTP joints Range of Motion (ROM) and recurrence of deformity were assessed independently by two Authors (T.G., C.P.). Secondary outcomes were presence of surgical complications and need for revision surgery.

Qualitative data were expressed as numbers with percentages, and quantitative data were expressed as means (±standard deviation) with a range.

Preoperative and final VAS, IP and MTP joints flexion ROM and AOFAS scores were compared using the Wilcoxon signed rank test. A *p*-value of <0.05 was considered statistically significant.

Statistical analysis was performed using SPSS 18.0 for Windows (SPSS Inc., Chicago, IL, USA).

All procedures were performed following written informed patient consent and in accordance with the ethical standards of the institutional and national research committee and the 1964 Declaration of Helsinki. Patients were not required to provide informed consent to process data for the study because the analysis used anonymous clinical data

The study was notified and discussed with Institutional Review Board of “Fondazione Policlinico Universitario Agostino Gemelli—IRCCS”.

The study design was approved by the Orthopedic Department council and our school board and has been reviewed for epidemiological and statistical validation by the public health institute of our institution.

A literature search was performed on MEDLINE through PubMed, Google Scholar and Web of Knowledge to identify publications concerning CD of the hallux or of the lesser toe. Literature search was performed on 28 February 2022, without applying any restriction on date of publication. To avoid missing studies, no filters were applied to the search strategies (Appendix B). Using titles and abstracts, two authors independently selected studies for inclusion. Studies with levels of evidence from I to V that recruited people of any age with CD of the hallux from any cause were included. Only papers published in English and with full-text available were considered for inclusion (PRISMA flowchart, Appendix B).

*Surgical technique*.

**Figure 2:**
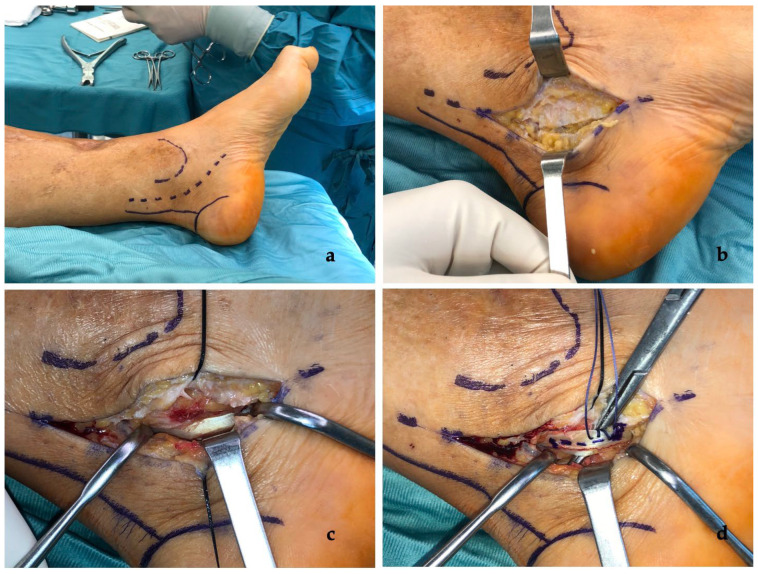
Case no. 4 in the series. (**a**) The retro-malleolar curvilinear approach to the tarsal tunnel. (**b**) Incision of the flexor retinaculum. (**c**) Exposition of flexor hallucis longus (FHL) tendon after opening of tendon sheath. (**d**) The FHL tendon Z-plasty sutured using 2-0 Ethibond.

Surgery was performed in supine position and under general or peripheral anesthesia. Antibiotic prophylaxis was administered, and then pneumatic tourniquet was applied to the thigh. Lower extremity was prepared, and a sterile field set up. A curvilinear retro-malleolar incision along the tarsal tunnel was made (Figure 2a). The flexor retinaculum was exposed to access the tarsal tunnel (Figure 2b); nerve, artery and posterior tibial vein were isolated and protected. FHL tendon sheath was identified and opened to expose about 3 cm of the tendon (Figure 2c). Tendon Z-plasty was performed and the first and second toes were passively extended; subsequently, with the ankle located 90° and the first and second toes in a neutral position, Z-plasty of the FHL tendon was completed using the 2-0 Ethibond (Figure 2d). After removal of tourniquet and checking for hemostasis, the skin was closed with 2-0 Ethilon.

After surgery was applied a compression bandage and the ankle was placed in neutral position in a Walker-type brace. After one month, the brace was removed and walking was allowed with progressive body weight loading.

## 3. Results

A total of 14 patients (8 males and 6 females) (Table 1) with a mean age of 37.4 ± 14.6 (range 19–63) were enrolled in the study.

The mean time from injury to onset of deformity ranged from 1 to 12 months with a mean of 7.0 ± 3.2 months.

Patients came to our attention complaining of pain and walking deficit (preoperative mean VAS 8.0 ± 0.7; preoperative mean AOFAS 39.7 ± 9.4) with inability to actively straighten deformed joints. In all patients, the first ray (big toe) was involved, in two patients the second toe was also involved and, in another two, both the second and third toes were involved. No neurological deficits were found on examination.

The mean follow-up of the patients included in the study was 31.8 ± 10.8 months, ranging from 18 to 48 months.

All patients were suffering from post-traumatic CD, with a history of tibial and/or fibular shaft fractures (n = 5), distal tibial and fibular fractures (n = 3), bimalleolar ankle fractures (n = 2), tri-malleolar ankle fractures (n = 2) or tibial plafond fractures (n = 2). Four of them also developed compartment syndrome. For the initial fracture treatment, seven had been treated with open reduction and internal fixation (ORIF) with plates and screws, five had undergone intramedullary nailing (IMN), and two with external fixation (EF) and ORIF with plates and screws (Table 2).

**Figure 3:**
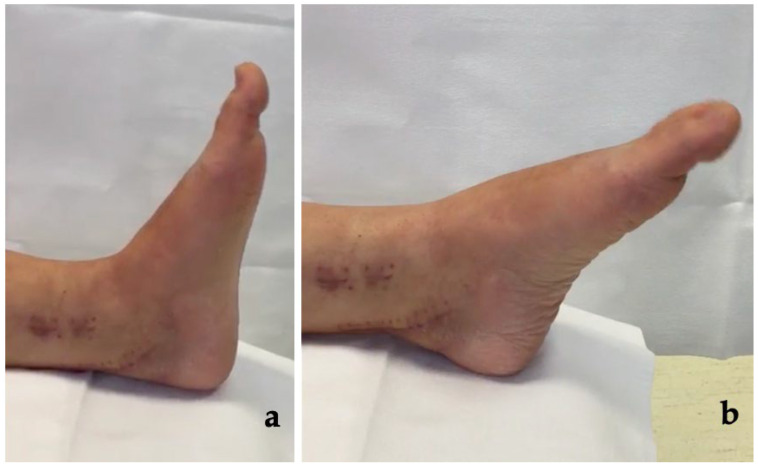
Post-surgery clinical images of case no.6 in the series. (**a**) Correction of deformity at dorsiflexion and (**b**) at plantar flexion of ankle.

After surgery patients experienced improvement of rigid IP joint flexion and rigid MTP contracture of the first toe (Figure 3, see Supplementary Material Video S2); passing from a mean preoperative flexion of the IP joint of 64.0° ± 7.2° to a mean postoperative IP flexion of 11.4° ± 4.1° (*p* < 0.05) and from a preoperative mean MTP joint flexion ROM of 12.1° ± 4.6° to a postoperative mean value of 36.0° ± 4.0° (*p* < 0.05) (Table 1 and Table 3).

A statistically significant improvement was also found for pain and postoperative function, mean postoperative VAS 1.1 ± 0.8 and mean postoperative AOFAS of 84.2 ± 8.5, respectively, (for both *p* < 0.05).

In patients with associated deformities in other toes, lengthening of FHL tendon with retro-malleolar Z-plasty and subsequent correction of the first toe also resulted in correction deformity of the other toes due to the interconnections between FHL and FDL.

All patients underwent successful surgical correction with no recurrence, no loss of strength, no nerve injury or tarsal tunnel syndrome. No patient required surgical revision.

## 4. Discussion

CD is a rare condition; 55 cases, with a predilection for the male sex, (42 vs. 13) have been reported in the literature. The mean age of onset is 27.8 years old (range 14–64 years old) (Table 4).

Deformity is mostly secondary to FHL tendon entrapment after a trauma with tibial, leg or ankle fracture, regardless of the internal fixation device used [3,4,5,8,28,29], as demonstrated also in the present series.

FHL arises from the inferior two-thirds of the posterior surface of the fibula and interosseous membrane, and it inserts into the plantar surface of the great toe distal phalanx. It allows to plantarflex the great toe and to supinate and plantarflex the ankle [6]. Feeney et al. [5] suggest that an explanation of the dynamic deformity could be the development of a subclinical compartment syndrome. An increase in the compartment might result in an injury to the musculotendinous junction of the FHL, with a slight shortening of the tendon due to hypo-atrophy and subsequent development of a dynamic deformity.

An average time of onset of deformity with respect to the initial trauma is difficult to calculate from the data available in the literature, ranging from a few days after the trauma [14,17] up to 60 months [2].

In the literature, treatment adopted was surgical in almost all cases. Only in one study [17], a 28-year-old female patient who underwent mandibulectomy for ameloblastoma and fibula resection for subsequent grafting, was treatment only with physical rehabilitation therapy. She underwent rehabilitation therapy for 20 days, including shock wave therapy and manual stretching therapy, and obtained an evident improvement in pain in all toes and gait stability, as well as increased ability to climb up and downstairs.

For surgical treatment various options have been proposed, but the optimal approach site and procedure for the correction of claw toe deformity is unclear. The two commonest procedures reported in literature for deformity correction are adherence release at the fracture site and FHL tendon lengthening (retro-malleolar or at the midfoot), variously associated. Adherence release alone can improve movement, but cannot correct hallux deformity, so should be associated with tendon lengthening to correct deformity [2,6]. Furthermore, in two case reports FHL tenotomy, at midfoot or at IP joint, was used [2,13].

Bai et al. [13] described a case of a 32-years-old man with ankle fracture dislocation combined with syndesmosis injury. Tibial and fibular fractures were treated with plates and screws and tibiofibular syndesmosis was stabilized with two suture-button devices. Two months after internal fixation, patient developed hallux disfunction due to entrapment of FHL muscle belly, due in turn to proximal misplaced suture-button. He underwent device removal, FHL tenotomy at midfoot, proximal to master knot of Henry, and suture between distal part of FHL and FDL to enhance flexion force of the great toe. Four weeks after surgery, patient achieved complete resolution of CD and, at 24 months follow up, no recurrence. Holcomb et al. [2], instead, in a 19-years-old man with distal tibial fracture associated with CD involving the first three toes, used flexor tenotomies on plantar aspect of the first, second and third toes IP joint. Furthermore, to keep correction, an interphalangeal arthrodesis of the hallux with a cannulated screw was made. Joint fusion in anatomic position achieved reduction, with no recurrence of the deformity and no complications.

Several studies propose a midfoot approach. In Sinnet et al. [7] and Gadhavi et al. [9] case reports analysed young male patients who complained of clawing great toe after distal tibial fracture and ORIF. Exploration of FHL tendon and lengthening with Z-plasty were performed at midfoot because the authors believed that there was easier access to both tendons, free from scar tissue. At three months follow up, patients maintained full range of movement in great toe and no recurrences were observed. Yuen and Lui [6], instead, evaluated a 36-years-old man with CD involving first three toes and treated with midfoot FHL release, after which the second and third toes’ clawing were corrected, but there was residual flexion deformity over the hallux. Additional Z-lengthening of the FHL tendon was performed at the segment between the adhesion site and the flexor retinaculum, correcting big toe deformity. At 17 months follow-up after the second surgery, there was no recurrence.

Lee and Kim [8], in the largest study currently present in the literature, analysed 11 patients suffering from CD (in five patients the first three toes were involved, in another five the first and second toes, and in one of them only the first), due to ankle or leg fracture or compartment syndrome, treated with two different approaches: six had FHL tendon Z-plasty in the midfoot and five underwent adherence release and tendon Z-plasty at the musculotendinous junction at the fracture site with a posteromedial approach. After a mean follow-up of 20,4 months, they observed no recurrence of the deformity in the six patients with tendon Z-plasty at the midfoot; instead, of the five patients with adherence release and tendon Z-plasty above the ankle, two showed partial and one a complete recurrence of the deformity. Patients with partial recurrence showed gradual improvement and did not wish to have a further operation. The patient with a complete recurrence had a further lengthening by Z-plasty on the medial side of the midfoot which resulted in complete resolution.

Retro-malleolar approach to the tarsal tunnel has been proposed for treatment of CD resulting from different types of fracture.

The largest study available in the literature relating to this approach is that conducted by Lee et al. [1], in which eight patients with CD of the first and second toes underwent lengthening of the FHL tendon alone or in combination of FHL and FDL tendons. After a mean follow up of 3,4 years, a mean tendon lengthening of 1.7 cm was observed, with complete correction of deformity, improvement in pain and gait shown by AOFAS score and no deformity recurrence. Moreover, lengthening of the FHL tendon alone may resolve deformity in the first and second toes

Kim et al. [15] observed CD after talus fracture. MRI showed the FHL trapped between talus fracture fragments. Surgical exploration was performed on the posteromedial aspect of the ankle 8 weeks after the initial injury. FHL was exposed and was freed from its entrapment; tenorrhaphy and reinforcement of the ragged portion of the tendon with suture and Guardix Solution^®^ was performed. Two months postoperatively, plantarflexion of the hallux was nearly normal.

Rosenberg and Sferra [11] found CD in a 14-years-old male following a closed Salter-Harris Type II distal tibia fracture with a distal third fibular shaft fracture proximal to the epiphysis, treated with closed reduction and cast immobilization. Seven months after initial injury he complained of flexion deformities in his great toe and second toe. FDL and FHL tendons were explored through a retro-malleolar approach and were found embedded in the scar tissue. Tendons were freed completely of adherences. Since a tibialis posterior (TP) tendon rupture was found too, a tenodesis between TP and FDL tendons just proximal to the medial malleolus was performed. Clinical examination and function of the ankle, hindfoot and forefoot were significantly improved after surgery.

Sanhudo and Lompa [10] presented two cases of CD. The first developed deformity after removal of a distal fibula graft for hip surgery, and the second developed a flexion contracture of the hallux, second and third toe after surgery for Weber C ankle fracture. The first case was treated by release and tendon lengthening with a retro-malleolar approach. One year after surgery, this patient was able to walk without pain although his toe extension was not complete. The second case was treated by lengthening of the FHL at the midfoot. Eight months after surgery this patient was able to walk without pain and regained good ROM.

Rodriguez-Collel and Mifsut-Miedes [20] reported the case of a 36-years-old male who developed CD of the hallux and of the second toe following a direct soft tissue trauma to his leg, with no associated fracture. Deformity correction with a retro-malleolar FHL tendon Z-plasty was performed, without the need for accessory surgical maneuvers to correct the other fingers. After surgery, the patient was immobilized in a splint for 3 weeks, with the ankle in neutral position. Following a short rehabilitation program (2 weeks), patient made a full recovery, with full restoration of active flexion/extension of the great and lesser toes.

Literature demonstrates how both approaches (retro-malleolar and midfoot) were effective in correcting deformity and relieving symptoms of fixed toe flexion.

Although some authors prefer FHL tendon lengthening to the midfoot due to the lower risk of adherence recurrence [7,10], with this approach an additional surgical time is required to separate FDL tendon attached to FHL tendon because the inter-tendinous connection in the midfoot does not allow correction of the deformity of the other toes [8].

On the other hand, the retro-malleolar Z-plasty lengthening, in the face of a greater risk of recurrence due to adherences and the need for meticulous dissection of the soft tissues to prevent injury to the neurovascular bundle, allows a simultaneous correction of the deformities on the big toe and on the other toes and lead to more powerful walking [1,5].

The present study has several limitations: the retrospective design of the study, the lack of a control group with another treatment and the small number of patients treated due to the rarity of the disease. Despite this, it is currently the largest case series in the literature and with a relatively long follow-up. Good results obtained, despite few cases in our series, allow consideration of new possible treatment options, but it is always necessary to consider the surgeon’s experience in choosing a surgical approach. In the next few years, it is expected that other clinical and surgical trials will be conducted to provide a more standardized approach for this disabling disease.

## 5. Conclusions

CD is an uncommon condition, but it must be known about and always considered as a possible chronic complication both in the soft tissue injuries of leg, and especially in ankle and tibial shaft fractures, so as not to misdiagnose.

Our case series and the available literature highlight how retro-malleolar (at the tarsal tunnel) FHL tendon Z-plasty represents a valid option for the correction of CD, allowing a good clinical and functional recovery with a low risk of recurrence and surgical complications.

## Figures and Tables

**Table 1:** Results.

**Number of Patients**	14	
**Sex**	8M, 6F (57.1%; 42.9%)	
Age *	37.4 ± 14.6 (range 19–63)	
Onset of Deformity * (months after trauma)	7.0 ± 3.2 (range 1–12)	
**Affected Toes** 1 1, 2 1, 2, 3	10 2 2	
**VAS** Pre * Post *	8.0 ± 0.7 1.1 ± 0.8	***p* < 0.05**
**Hallux MTP Flexion** Pre * Post *	12.1 ± 4.6 36.0 ± 4.0	***p* < 0.05**
**Hallux IP Flexion** Pre * Post *	64.0 ± 7.2 11.4 ± 4.1	***p* < 0.05**
**AOFAS Score** Pre * Post *	39.7 ± 9.4 84.2 ± 8.5	***p* < 0.05**
Follow-Up * (months)	32.0 ± 10.6 (range 18–48)	

M: Male; F: Female; VAS: Visual Analogue Scale; MTP: Metatarsophalangeal; IP: Interphalangeal; AOFAS: American Orthopedic Foot and Ankle Society; Pre: Pre-surgery; Post: Post-surgery. * mean ± SD (standard deviation).

**Table 2:** Demographic characteristics and clinical features of patients.

No.	Sex	Age	Etiology	First Treatment	Onset of Deformity (Months after Trauma)	Affected Toes	Follow-up (Months)
1	M	50	Tibial and fibular shaft fracture	IMN	10	1	36
2	F	25	Open distal tibial and fibular fracture #	EF + Plate and screws	8	1, 2	20
3	F	32	Bimalleolar ankle fracture	Plate and screws	10	1	42
4	M	63	Tibial and fibular shaft fracture	IMN	7	1	24
5	M	21	Tri-malleolar ankle fracture	Plate and screws	9	1	48
6	M	19	Tibial and fibular shaft fracture	IMN	2	1	24
7	F	53	Tibial plafond fracture #	Plate and screws	1	1, 2, 3	36
8	M	28	Tri-malleolar ankle fracture	Plate and screws	5	1	36
9	M	39	Tibial and fibular shaft fracture	IMN	8	1	20
10	F	47	Distal tibial and fibular fracture #	EF + Plate and screws	10	1, 2	48
11	M	33	Distal tibial and fibular fracture	Plate and screws	6	1	18
12	F	20	Open tibial shaft fracture	EF + IMN	8	1, 2, 3	42
13	F	36	Tibial plafond fracture #	Plate and screws	12	1	32
14	M	58	Bimalleolar ankle fracture	Plate and screws	3	1	22

M: Male; F: Female; IMN: Intramedullary nailing; EF: External Fixation # Compartment syndrome.

**Table 3:** Comparison of VAS score, ROM and AOFAS score pre- and post-surgery.

No.	VAS		Hallux MTP Flexion (°)		Hallux IP Flexion (°)		AOFAS Score	
	Pre	Post	Pre	Post	Pre	Post	Pre	Post
1	9	2	10	35	70	10	47	90
2	8	1	15	40	66	15	24	72
3	7	2	15	35	65	10	37	82
4	9	0	10	40	75	5	42	92
5	7	1	5	30	60	10	32	97
6	8	2	20	35	70	15	47	67
7	9	0	10	40	65	15	52	77
8	8	1	15	35	60	10	34	79
9	8	0	10	30	45	10	38	85
10	8	2	10	40	63	5	47	90
11	9	2	20	40	60	10	32	88
12	8	2	5	30	72	20	57	79
13	8	1	15	35	60	15	40	90
14	7	0	10	40	65	10	28	91

VAS: Visual Analogue Scale; MTP: Metatarsophalangeal; IP: Interphalangeal; AOFAS: American Orthopedic Foot and Ankle Society; Pre: Pre-surgery; Post: Post-surgery.

**Table 4:** Literature analysis.

Authors, Year	Number of Cases	Sex	Age	Etiology	Fingers Involved	Time Trauma to Deformity (Months)	Treatment	Follow-Up (Months)
Carr [14] 1990	2	F	21 35	Calcaneus Fracture	1	Within 5 days	Tendon dislocation reduction	30 24
Leitschuh et al. [12], 1995	1	M	61	Weber C ankle fracture	1	18	Release + medial tenodesis	NS
Rosenberg and Sferra [11], 1999	1	M	14	Salter-Harris II distal tibia fracture and distal fibular shaft fracture	1, 2	1,5	Release + retro-malleolar tenodesis of tibialis posterior to FDL+ great toe IP and second toe PIP capsulotomies	NS
Feeney et al. [5], 2001	10	8M-2F	27 *	8 tibial shaft fracture; 1 post-traumatic compartment syndrome; 1 prolonged lithotomy positions with ankle strips	(1) 2–5 (2) 1 (2) 1–3 (2) 1–4 (3) 1–5	6	FHL/FDL tendon lengthening with Z plasty	12
Sanhudo and Lompa [10], 2002	2	1M-1F	45 31	Distal fibular graft for hip surgery; Weber C ankle fracture	1	2 1.5	(I) release + retro-malleolar tendon lengthening (II) FHL and FDL lengthening at midfoot	NS
Lee and Kim [8], 2008	11	6M-5F	36 *	Bimalleolar, tri-malleolar, pilon, biosseous leg fracture, soft tissue injury without fracture	(5) 1 (5) 1–2–3 (1) 1–2	3.5 *	(6) midfoot lengthening of FHL by Z plasty(+2 weeks with short removable splint) (5) release and musculotendinous Z-plasty lengthening	20.4 *
Kim et al. [15], 2010	1	M	44	Ankle sprain with talar fracture	1	2	fracture reduction+ tendon release with posteromedial approach	2
Holcomb et al. [2], 2014	1	M	19	Distal tibia fracture	1–2–3	60	FHL tenotomy over the IP joint + hallux IP joint arthrodesis	12
Sinnet et al. [7], 2015	1	M	22	Distal tibia and fibula fracture	1	48	Midfoot FHL tendon Z-plasty	3
Abolfotouh et al. [16], 2015	3	M	40 36 30	Intra-articular talar fracture	1	0	Fracture reduction + tendon release	24 24 18
Yuen and Lui [6], 2015	1	M	36	Tibial and fibular shaft fracture	1–2–3	6	Rehabilitation therapy first, FHL tendon Z-plasty with posteromedial approach to the distal tibia	17
Tanwar et al. [19], 2016	1	M	26	Peri-talar dislocation	1	0	Dislocation reduction + stabilization with 2 Kirschner wires across subtalar joint and 1 across talonavicular joint	NS
Lee et al. [1], 2016	8	6M-2F	39,5 *	Tibiofibular, distal fibular fracture and crush injury around the ankle	1–2	8.4 *	Retro-malleolar FHL tendon Z-plasty	3.4 *
Park et al. [27], 2017	5	M	34,8 *	2) laceration on great toe 3) IP joint dislocation	1	1.5 *	Midfoot FHL tendon Z-plasty	44
Gadhavi et al. [9], 2019	1	M	25	Distal tibia fracture	1	24	Midfoot FHL tendon Z-plasty	3
Bai et al. [13], 2020	1	M	32	Ankle fracture-dislocation combined with syndesmosis injury	1	2	Removal of the suture-button device + midfoot FHL tendon tenotomy and suture of distal part of the FHL to the FDL	24
Feng at al. [17], 2020	1	F	28	Fibular grafting for mandibular ameloblastoma	1–2–3	20 days	Physical rehabilitation therapy	6
Sallent et al. [18], 2020	2	M	27 36	Fibular free flap for mandibular mesenchymal chondrosarcoma/squamous carcinoma	1–2	NS 6	Hallux arthrodesis + midfoot FHL tendon Z-plasty and FDL tendon tenotomy	NS 5
Rodriguez-Collell and Mifsut-Miedes [20],2021	1	M	37	Soft tissue trauma (compartment Syndrome)	1–2	12	Retro-malleolar FHL tendon Z-plasty and application of a pulvertaft suture to the flexor hallucis longus	NS
Greco et al. [3], 2021	1	M	63	Open tibia and fibula fracture	1	7	Retro-malleolar FHL tendon Z-plasty	24

M: Male; F: Female; NS: Not specified; FHL: Flexor hallucis longus; FDL: Flexor digitorum longus; IP: Interphalangeal; PIP: Proxiaml Interphalangeal. * mean.

## Data Availability

The study data will be available upon request to the corresponding author (email: greco.tommaso@outlook.it).

## Supplementary Materials

The following supporting information can be downloaded at: https://www.mdpi.com/article/10.3390/medicina58081072/s1, Video S1: Pre-surgery clinical presentation, Video S2: Post-surgery clinical presentation.

## Appendix A

STROBE checklist.

## Appendix B

Search strategies and PRISMA Flowchart.
